# Burnout syndrome in resuscitation anesthesiologists as a subjective answer to professional stress in the covid-19 period

**DOI:** 10.1192/j.eurpsy.2022.1252

**Published:** 2022-09-01

**Authors:** L. Baranskaya, A. Kolesnik, A. Levitt

**Affiliations:** 1 Ural State Medical University, Psychiatry, Psychotherapy And Narcology, Yekaterinburg, Russian Federation; 2 Ural State Medical University, Psychiatry, Psychotherapy Fnd Narcology, Yekaterinburg, Russian Federation; 3 Ural State Medical University, Anesthesiology And Reanimatology, Yekaterinburg, Russian Federation

## Abstract

**Introduction:**

In the COVID-19 pandemic situation the burden that has fallen on the shoulders of resuscitation anesthesiologists has become really great. These specialists experience emotional and psychical stress that has led to the high risk of formation of the emotional burnout syndrome in the pre-COVID period.

**Objectives:**

A comparative analysis of the formation of a complete syndrome of emotional burnout in 2020-2021 in resuscitation anesthesiologists with varied years of professional activity.

**Methods:**

Sixty-two resuscitation anesthesiologists volunteered to take part in the study: 47 males and 15 females. The main method of study was V. Boiko’s method of “Diagnosis of level of emotional burnout”.

**Results:**

The results have shown that, during the said period, the number of doctors with complete syndrome of emotional burnout has significantly increased, that is, all three phases: stress, resistance and exhaustion had formed. The period of study has clearly shown two groups of male doctors: with period of work of 20 or more years, and with period of work of less than 5 years. The said symptoms cause a feeling of physical and psychological overburdens, stress at work and at home, conflicts with management personnel, colleagues and patients.

**Conclusions:**

The atypical COVID-19 pneumonia pandemic has laid significant stress on the psychic and physical health of resuscitation anesthesiologists. The high level of psychological strain, accumulation of negative emotions, and the feeling of helplessness led to medical errors and delays in important tactical decisions.

**Disclosure:**

No significant relationships.

